# Neoteric approach for peanuts biofilm using the merits of Moringa extracts to control aflatoxin contamination

**DOI:** 10.1016/j.toxrep.2021.08.006

**Published:** 2021-09-10

**Authors:** Yehia Hassan Abu-Sree, Shaaban Mostafa Abdel-Fattah, Adel Gabr Abdel-Razek, Ahmed Noah Badr

**Affiliations:** a Food Toxicology and Contaminants Department, National Research Centre, Dokki 12622, Cairo, Egypt; b Fats and Oils Department, National Research Centre, Dokki 12622, Cairo, Egypt

**Keywords:** Biofilm emulsion, Leaves extract, Moringa oil, Peanut protection, Toxigenic fungi

## Abstract

Aflatoxigenic fungi and aflatoxins are still a principal challenge that threatened peanut production, marketing, and handling. This study aimed to face the problem using bioactive materials, which reduce fungi and mycotoxin contamination, Moringa extracts may be suitable for solving this challenge. Also, the study was compared the extracts of leaves and oil-free seeds. Fresh leaves and seeds were collected, dried, and milled, while oil was collected by cold pressing. The extracts were evaluated for total phenols, flavonoids, and antioxidants, the oil contents of fatty acids, tocopherol, and sterols were determined. An emulsion for protecting peanuts compositing of leaves extract carried by Moringa oil, and commercial emulsifier. Leaves extract evaluation reflected distinct properties of its fibers, total phenols, and flavonoids. It was recorded a microbial inhibition of bacteria and fungi. The values ​​for both minimal inhibition and fungicidal concentrations were recorded at 3.2 mg/mL and 490 μg/L, respectively. For oil, it showed a unique content, as oleic acid was the main fatty acid, with an affinity between palmitic and behenic in their ratios. Also, oil was recorded by high contents of alpha-tocopherol and Δ7-Campesterol, with 1.166 mg/kg oil as total sterols content. The leaves extract has also a unique capacity to inhibit toxigenic fungi. By applying the composite emulsion for peanut coating, results expressed a high CFU-count inhibition when it was inoculated by *A. flavus* strain compared to the control.

## Highlights

• The Moringa leaves extract is rich in minor components.
• The Moringa oil distinct content of tocopherols & sterols contents.
• Moringa leaves extract possessed anti-mycotic and antiaflatoxigenic characteristics.
• The Oil/Water film was effective to protect peanuts against aflatoxin.

## Graphical abstract

**Figure:**
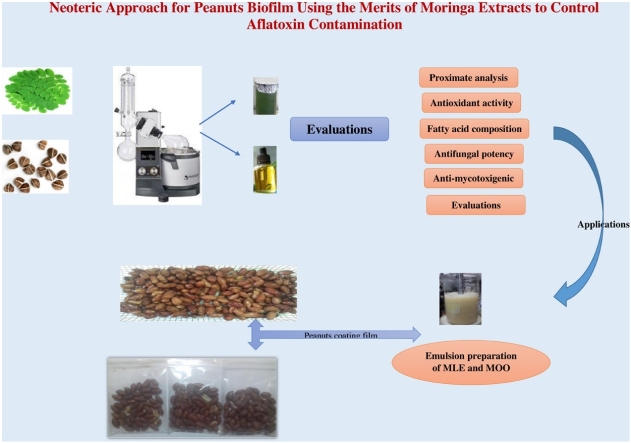

## 1 Introduction

Food manufacturers are more responsible for producing fresh and organic ingredients, which are still safe to eat. They try to use synthetic chemicals for preventing foodborne diseases as little as possible. The drug/chemical resistance turns this action to be none preferable [1]. Plant seeds have natural defines system, which offers the first line of protection against spoilage [2]. This system contains minor components that play double functions, nutritional and prevention. Phenolic, tocopherols, antioxidants, peptides, and fibers are the principal bioactives that participate in performing these functions [3,4]. It also acts like natural antimicrobial agents, which are leading to higher food safety standards with preventing microbial infection [5,6]. The antimicrobials constituents are involving antioxidants, phenolic compounds, flavonoids, as well active peptides [[7], [8], [9]]. The unique characteristic of Moringa extracts is the capacity to perform all these roles due to their bioactive and functional constituents.

*Moringa oleifera* is palpably disseminated, where it can adapt to climatic and soil variations [10]. It is a highly beneficial plant and all pieces can utilize in food composites [11,12]. Moringa named a living tree, which originated name due to functionality applications, basically in medicine and nutrition [13,14]. Moringa extracts are a valuable bioactives source that is utilized in food applications [15,16]. Moringa has high oil content besides functional carbohydrates, proteins, and specific polypeptides [13,17]. The seeds are recognized in food and non-food applications due to their content of fatty acids [18], sterols, tocopherol, as well as, essential amino acids [19].

Notwithstanding, toxigenic fungi are critical hazards that are facing food production. It is caused serious problems in agricultural commodities, particularly aflatoxins and ochratoxins in cereals, grains, and nuts [5,20]. Bioactive components represented an efficacy for fungal inhibition and mycotoxin reduction. These bioactives, which are applied in food manufacturing, could be sourced microbiologically as metabolites [21,22], or as plant extract [7,23,24]. The bioactive carbohydrates including dietary fibers have a positive impact as antimycotic and anti-mycotoxigenic agents against the toxigenic fungi activities on food commodities [[25], [26], [27]]. Recently, several investigations referred to plant extract to perform antifungal and anti-mycotoxigenic properties [7,28]. These extracts are included polar types [5,23] and non-polar types [29,30] of extract. Vegetarian oils, particularly the non-traditional kinds, represented a unique bioactive source effective versus fungi and their toxins [6,8]. Where the Moringa plant is known rich in minor constituents, it prospects to provide valuable action for the biological evaluation.

Numerous studies were handling the antimicrobial impact of Moringa, but limited studies determined its antifungal activity. Moreover, usually peanuts are coated using salt or chocolate for flavoring, without any attention for the possibility of aflatoxin contamination. The study presented a neoteric approach that is achieving two targets; peanut coating and protection. The implementation for two types of Moringa extracts to form coating film that provides prevention against both hazardous toxigenic fungi and their related mycotoxin, as well as it could be loaded by flavors. As an application, Moringa emulsion consisted of Moringa leaves extract (MLE) in Moringa oil (MOO), emulsified by a commercial emulsifier, was utilized for protecting against aflatoxigenic-fungi inoculation of peanut. Concerning forward of this, the study was determined the chemical, biological, and nutritional characteristics of extracts. Also, the study was referred to the bioactive constituents and their valuable quantities.

## 2 Materials and methods

### 2.1 Raw materials and chemicals

Leaves and seeds of *Moringa oleifera* were purchased from the botanical herbarium garden of the National Research Centre (special unit) as fresh collecting materials kept at 2℃during the analysis procedures.

The microbial strains of bacteria were *Bacillus cereus* ATCC 4342; *E. coli* ATCC 11229; *Staphylococcus aureus* NCTC 10788; *Salmonella typhi* ATCC 14028 purchased from Egyptian Microbial Culture Collection (EMCC), Ain Shams, Egypt. The strains of toxigenic fungi applied in this experiment were *Aspergillus flavus* ITEM 698, *A. ochraceus* ITEM 5010, *A. niger* ITEM 3856, *A. fumigatus* ATCC 1022 *Fusarium verticillioides* ITEM 674, *Penicillium chrysogenum* ITEM 5152, *Alternaria alternata* ITEM 752 obtained from Agrofood Microbial Culture Collection (ITEM), ISPA, Italy.

### 2.2 Leaves and fat-free seeds preparation

The leaves were dried by the hot-air oven (ShelLab-Model 1350FX-Sheldon manufacturing, INC, the USA) at 40 ± 3 °C/36 h, ground to powder, and milling using a locally Milling machine (mini mill micro grinder plant machine RH™), the powder sieves through 40 mesh. Leaves powder were extracted using aqueous-methanol (80 %), evaporated using rotary-evaporator, and kept dried for further application. The uniform seeds were defatted using a mechanical press machine to obtain the fat-free seeds (MFFS), where the oil was collected separately. The investigated non-polar extracts of powders were prepared using petroleum ether (40:60), then evaporated (Heidolph, Hei-VAP Platinum 5; ProfiLab24 GmbH; Landsberger; Germany) until dryness, where it was re-dissolved in dimethyl sulfoxide (DMSO) for microbial examinations.

### 2.3 Proximate analysis

Carbohydrates, moisture, fat, protein, ash, and fiber contents were determined according to the AOAC methodology described by Sultana [31]. The carbohydrate content was determined by difference, after the addition of all the percentages of moisture, fat, crude protein, ash, and crude fiber, and was subtracted from 100 %. The contents of neutral detergent fiber (NDF), acid detergent fiber (ADF), acid detergent lignin (ADL), hemicellulose, cellulose, and lignin were determined by using Fiber tec system VELP 014_EN fiber extractor 3–6 posts Fisher Bio-block Scientific [32].

### 2.4 Fatty acid analysis of Moringa oil

The fatty acid content of the MOO was determined according to the methodology described by Abdel-Razek et al. [6], by the same conditions using the GC–MS apparatus. Briefly, oil was analyzed by Agilent 7890 apparatus (Agilent Technologies, Santa Clara, CA, USA) supported by the FID and capillary Innowax column (30 m × 0.20 mm × 0.20 mm). The flow rate of carrier gas was 1.5 mL/min, and the column temperature was 210 °C. The results were recorded as weight percentages after integration and calculation using the Chem-Station and comparing the retention times with authentic standards.

### 2.5 Total phenolic content determination (TPC)

The total phenolic contents of the Moringa parts were evaluated using the recommended method by the European Pharmacopoeia that was described by Blainski et al. [33]. The results are expressed as mg Gallic acid equivalent (GAE)/100 mL of the crude extract. The purified phenolic extract was vacuum-evaporated to near dryness by the rotary (35 °C), finally dissolved by aqueous methanol. About 0.2 mL of extracted solution and diluted Folin–Ciocalteu’s phenol reagent (1 mL) were mixed. After 3 min, sodium carbonate (0.8 mL/7.5 %) was added, the mixture was rested for 30 min, and absorbance was measured using a UV spectrophotometer (Shimadzu, Kyoto, Japan) at wavelength 760 nm. The TPC was measured and expressed as mg Gallic acid equivalent (GAE)/100 g of the crude extract against the blank.

The total flavonoid content (TFC) of the Moringa extracts was determined using an aluminum chloride colorimetric method as described before [34]. Briefly, 1 mL aqueous methanol extract (1 mg/mL; 80 % methanol) or standard catechol solution was mixed with one milliliter of the AlCl_3_ (2% w/v) in methanol. The absorbance against blank was measured at 440 nm using a UV spectrophotometer (after incubating for 40 min/25 °C). The TFC was calculated using the catechol calibration curve; results were expressed as catechol equivalent (CE) per gram of dried powder, where the samples were performed in triplicate.

### 2.6 Antioxidant activity estimation

The free radical scavenging capacity of extracts was determined using the stable DPPH by the same method mentioned by Shehata et al. [30]. The process is based on the DPPH (1, 1-diphenyl-2-picrylhydrazyl) radical scavenging hypothesis. The DPPH was applied to the extracts or the standard antioxidant compounds within solutions, which were then stirred. The absorbance of each mixture was evaluated at 517 nm versus the blank after half an hour in darkness.

The free radical-scavenging activity of samples was determined by ABTS radical Cation decolorization assay [30]. The stock solutions (7 Mm ABTS solution) and potassium per-sulfate solutions. The working solution was prepared by mixing these stock solutions in equal proportions and allowing them to react (12−16 h) in darkness. A control containing methanol and ABTS^•^+ solution was also realized. The absorbance was read at 734 nm after 30 min incubation at 25 °C. The percentage inhibition of free radical ABTS was calculated.

Ferric reducing antioxidant power also applied for the antioxidant capacity was also estimated spectrophotometrically following the procedure described by Rajurkar and Hande [35]. The method is based on the reduction of Fe^3+^ TPTZ complex (colorless) to Fe^2+−^ tri-pyridyltriazine (blue) formed by the action of electron-donating antioxidants at a low pH value. This reaction is monitored by measuring the change in absorbance at 593 nm. An intense blue complex was estimated at 593 nm against the blank and expressed as mg of Trolox equivalent for each gram of sample. All the determinations were performed in triplicates.

### 2.7 Determination of tocopherol, Tocotrienol, and sterols fractions

The oil content of tocopherol and tocotrienol was determined using HPLC (Agilent 1100 apparatus) as described by Abdel-Razek et al. [6], using the same conditions. While, the sterol fractions of Moringa oil were analyzed according to the methodology of Stuper-Szablewska et al., [36], which was performed using an Aquity H class UPLC system equipped with a Waters Acquity photodiode array (PDA) detector (Waters, USA).

### 2.8 Determination of antimicrobial properties

The antimicrobial properties were determined using several assays to ensure its efficiency. Firstly, the minimal inhibitory concentration (MIC), minimal bactericidal concentration (MBC), and the minimum antifungal concentration (MFC) were evaluated as the lowest concentration affected the bacteria and fungi growth rates, respectively. The MIC was determined as the lowest concentration at which no growth occurs, it was done as a method described by Sakanaka et al. [37]. Azithromycin was applied as a positive control. Regarding the MFC, fungal-growth inhibition was tested to determine the impact of extracts as previously described by Abdel Razek et al. [28]. The concentration required to give 50 % inhibition of hyphen growth IC50 was calculated from the regression equation, where Mycostatin was used as a positive control. The antibacterial antifungal characteristics of extracts also were determined using the diffusion assay as the methodology described previously.

### 2.9 Determination of zone inhibition impact

The impact of Moringa extracts to represent zone inhibition against the fungi growth on solid media was determined regarding six toxigenic strains of fungi, four out of them were belonged to the *Aspergillus* genus. This experiment was done using the same methodology described by Shehata et al. [30]. Briefly, Autoclaved Potato dextrose agar was poured into sterile Petri dishes (15 cm), five-millimeter diameter wells were made in the four principal directions of the plate using cork-porer tools. Each well was filled with 100 μL extract and a paper disk of fungi spores was inoculated to the plate center. The plates were incubated (28 °C/96 h), where the zone diameter of inhibition was measured in millimeters.

### 2.10 Application of Moringa extracts to increase the Peanut safety

Peanut deems the great grains contaminated by aflatoxins, which affect the body tissues mainly the liver [38,39]. The polar extract (MLE) carried by non-polar extract (MOO) was applied to protect the grain against the contamination by toxigenic strains of fungi and their metabolites was a successful technique [30]. Briefly, methanolic extract (80 %) of Moringa leaves (ML) was loaded into MOO using 1% commercial emulsifiers (Panodan 150, Detam, DANESCO Co., India). The methanolic extract was loaded at a concentration of 5% of oil (contains 1% lecithin), and the produced emulsion was applied to coat the peanut seeds. The emulsion preparation was done according to the methodology of Abdel-Razek et al. [6]. An aflatoxin producer strain of *A. flavus* ITEM 698 was inoculated to three groups of investigated seeds, where the control one was not coated. The other two groups of grains were the red-peeled (shielded) and non- peeled (unshielded) seeds. Treated and control seeds were stored for 3 months, where the CFU counts of fungi were recorded each week. The final count after the storage period was considered the comparing agent, which reflects the protection effect of applied emulsion. The fungal-CFU counts were recorded during the incubation time using the serial dilution technique [40]. The less fungal-CFU counts, the more resistant seed of fungal contamination with less aflatoxin concentration.

### 2.11 Determination of aflatoxin concentration of coated peanut grains

Aflatoxin concentration of the control and coated grains after inoculation by the aflatoxin-producing strain were measured according to the methodology described by Abdel-Razek et al. [6]. The Aflatoxins determination was done using HPLC- Agilent 1100 apparatus (Agilent Technologies, Hewlett-Packard Strasse 876337 Waldbonn, Germany).

## 3 Results

### 3.1 The proximate analysis of Moringa fraction-parts

The analysis for Moringa fraction-parts manifested that carbohydrates content was recorded by high value in dry milled leaves but at approximately value quarter for the defatted seeds. While protein contents were recorded by more than double in fat-free seeds (Table 1). It is worth mentioning that the fiber content in leaves was approximately doubled its amount in seeds, while it still appears significant in both. This point leads the authors to analyze the fiber fractions to know the predominant fraction. The ash content of seeds powder was recorded higher than its value in dry-milled leaves powder. It was clearly shown that the defatted powder contents of protein and ash appeared higher compared to seed analysis, and these results are expected where the Moringa seed is a type of oilseeds.

**Table 1:** Chemical composition of different moringa plant parts.

Variables	% Carbohydrates	% Moisture	% Fat	% Protein	% Ash	% Fiber
Dried leaves	45.16^a^±0.72	5.51^a^±0.11	6.78^a^±0.12	27.46^b^±0.27	3.34^a^±0.12	7.85^a^±0.15
Fat-free seeds	10.48^c^±1.03	3.74^b^±0.18	4.85^c^±0.17	65.63^a^±0.51	8.41^c^±0.12	3.7^b^±0.13
L.S.D value	2.503	0.411	0.416	1.78	0.427	0.47

● The results are expressed as percentage means ± SD (n = 3).

● The least significant differences values for each (LSD) were represented in the lower row of each column.

### 3.2 Fiber fraction analysis of the Moringa leaves and seeds

The fractions of fiber content were analyzed to explore their contents and record the differences between the Moringa parts (Table 2). The contents of cellulose in leaves powder were recorded by a high value comparing to fat-free seeds powder, while the hemicellulose content was closely in leaves and fat-free seeds powder. The lignin content appeared higher in fat-free seeds than leaves, the same was shown for the acid detergent lignin ratio. Acid and neutral detergent fiber content were recorded with great values for leaves powder, while in defatted powder their values were recorded close to the bisection of their values in leaves.

**Table 2:** Fiber content of Moringa leaves and defatted seeds.

Variables	Leaves powder	Fat-free seeds
Cellulose%	7.96^c^±0.67	4.71^b^±0.74
Hemicelluloses%	7.55^c^±0.35	6.07^b^±0.27
Lignin%	1.97^c^±0.31	2.64^b^±0.34
Neutral Detergent Fiber (NDF)%	21.03^c^±2.08	11.39^b^ ±1.83
Acid Detergent Fiber (ADF)%	13.89^c^±1.23	7.84^b^±0.66
Acid Detergent Lignin (ADL)%	1.29^c^±0.55	2.11^b^±0.41

● The results are expressed as a percentage means ± SD (n = 3).

● The change in the superscription letters of each value expressed in each row represents significant differences at P=0.05.

### 3.3 The fatty acid content of Moringa oil

Moringa was a rich monounsaturated fatty acid oil (76.89 %), with oleic acid (73.61 %) being the most prevalent MUSFA (Table 3). The unsaturated fatty acid proportion was 78.27 percent, which appeared greater than the one reported by Sonntag (1982). Palmitic acid was the most prevalent saturated fatty acid (6.21 %), while total saturated fatty acids exceeded 21.72 % and minor levels (1.38 %) of polyunsaturated fatty acids were found in the oil.

**Table 3:** Fatty acid composition of moringa seed oil.

Component	% FAs
Caprylic (C8:0)	0.054 ± 0.033
Myristic (C14:0)	0.16 ± 0.028
Palmitic (C16:0)	6.21 ± 0.45
Palmitoleic (C16:1)	1.32 ± 0.11
Margaric (C17:0)	0.08 ± 0.029
Stearic (C18:0)	4.88 ± 0.15
Oleic (C18:1)	73.61 ± 2.12
Linoleic (C18:2)	0.64 ± 0.185
Linolenic (C18:3)	0.74 ± 0.157
Arachidic (C20:0)	3.1 ± 0.317
Gadoleic (C20:1)	1.82 ± 0.128
Behenic (C22:0)	5.97 ± 0.128
Erucic (C22:1)	0.14 ± 0.026
Lignoceric (C24:0)	0.3 ± 0.231
Cerotic (C26:0)	0.97 ± 0.102
Total Saturated FA	21.72
Mono Unsaturated FA	76.89
Poly Unsaturated FA	1.38
PUFA/SFA	0.064
USFA/SFA	78.27/21.72 = 3.6
Cox Value	(76.89 + 10.3*0.64 + 21.6*0.74)/100 = 0.99

● The calculated oxidizability (Cox) value of the oils was calculated by applying the formula proposed by Fatemi and Hammond [42], calculated as = [1(%C_16:1_+%C_18:1_+%C_20:1_)+10.3(%C_18:2_+%C_20:2_)+21.6(%C_18:3_)]/100.

The computed oxidizability (Cox value) was calculate to Moringa oil (Table 3) as described by Herchi et al. [41], ir was recorded as 0.99 in this study, compared to 2.23, 7.00, and 6.00 for olive, soybean, and sunflower oils, respectively, in prior work by Abdel-Razek et al., [42], whereas the PUFA/SFA was 0.064. The PUFA/SFA ratio and Cox value are commonly used to assess the proclivity of oils to oxidize. It was established that the fatty acid composition (Cox value) dictated the oils' oxidative stabilities, but that when Cox values were comparable, the tocopherol content of the oils would play a crucial role [43]. In light of the present result, Moringa oil manifested considerable stability that recommends it for the application in food coating-film of ready-to-eat products.

Moringa oil content of Tocols (tocopherol and tocotrienol) was appeared by moderate values, the oil contents of alpha and gamma-tocopherol were distinguished. The total content of tocopherols was about 32.06 mg/Kg oil, while a total content of tocotrienols was recorded at about 0.21 mg/Kg oil (Table 4). Notwithstanding, sterols content in Moringa oil was recorded totally as 1.116 ± 0.01 mg/Kg oil, by a majority for Campesterol fractions.

**Table 4:** The minor phytoconstituents content in Moringa oil.

Tocopherol fractions (mg/kg oil)		Tocotrienol fractions (mg/kg oil)		Sterols (mg/kg oil)	
α -tocopherol	11.66 ± 0.82	**α -tocotrienol**	0.11 ± 0.02	**Campesterol**	0.032 ± 0.001
β-tocopherol	1.67 ± 0.34	**β-tocotrienol**	ND	**Δ7-Campesterol**	0.791 ± 0.004
γ-tocopherol	18.96 ± 2.54	**γ-tocotrienol**	0.08 ± 0.001	**Stigmasterol**	0.221 ± 0.002
δ- tocopherol	4.77 ± 0.93	**δ- tocotrienol**	0.02 ± 0.005	**Δ7-Stigmasterol**	0.151 ± 0.001
				**β-Sitosterol**	0.109 ± 0.002
Total Tocopherol	32.06 ± 4.36	**Total Tocotrienol**	0.21 ± 0.008	**Total sterols**	1.166 ± 0.01

● The results are expressed as a percentage means ± SD (n = 3).

### 3.4 The determination of phytoconstituents of Moringa fractions

Total phenolic, total flavonoids, and antioxidant activities of the ML and fat-free seeds were determined to explore the phytoconstituents of Moringa parts (Table 5), also attempt to discuss their activities. The analyses will also consider a guide lead the antimicrobial explanation. The results statement that the MLE was recorded by distinguished concentrations of total phenolic and antioxidant activity. It is worth mentioning that the flavonoid of the ML was noticed by great content, which points out leaves as the principal flavonoid source in the Moringa plant.

**Table 5:** Phenolic, flavonoid, and antioxidant contents measured for moringa leaves and seeds.

Parameters (mg/g)	Leaves	Fat-free seeds
Total phenolic	12.39^c^±1.81	1.54^b^ ±0.77
Total flavonoids	7.68^c^±0.51	0.15 ^a^±0.08
DPPH scavenging	16.95^c^±1.48	0.57^b^±0.07
ABTS^+^ scavenging	14.17^c^±1.12	0.44^b^±0.03
FRAB scavenging	15.76^c^±1.53	0.49^b^±0.06

● The results are expressed as a percentage means ± SD (n = 3).

● The change in the superscription letters of each value expressed in each row represents significant differences at P=0.05.

### 3.5 Antimicrobial activity of Moringa extracts

The examination of Moringa extracts for their antimicrobial activity against pathogen strains of bacteria recorded the capacity of the MLE as more effective than fat-free seeds extract (Table 6). The MLE was recorded with minimal inhibitory and minimal bactericidal concentrations lower than seed extract, where they were compared to a standard antibiotic (Amoxicillin). It was noticed by narrow variation for its impact for gram-negative and gram-positive strains, which may be connected to the cell membrane components variation. But generally, the antimicrobial and antibacterial activities of the MLE may play a valuable function for food preservation.

**Table 6:** The minimal inhibition concentration of moringa leaves and fat-free seeds against six food-pathogen strains of bacteria.

Strains	Test name	Moringa leaves	Fat-free seeds	Amoxicillin (Standard antibiotic)
		Concentration (mg/mL)		
*Pseudomonase aerugnosa ATCC 9027*	MIC*	3.2	4.5	0.01
*Pseudomonase aerugnosa ATCC 9027*	MBC**	3.6	7.1	0.02
*Enterococcus faecalies*	MIC	3.7	5.4	0.02
*Enterococcus faecalies*	MBC	3.9	9.1	0.05
*Streptococcus* sp.,	MIC	3.4	3.2	0.03
*Streptococcus* sp.,	MBC	3.7	5.4	0.05

**Fig. 1:**
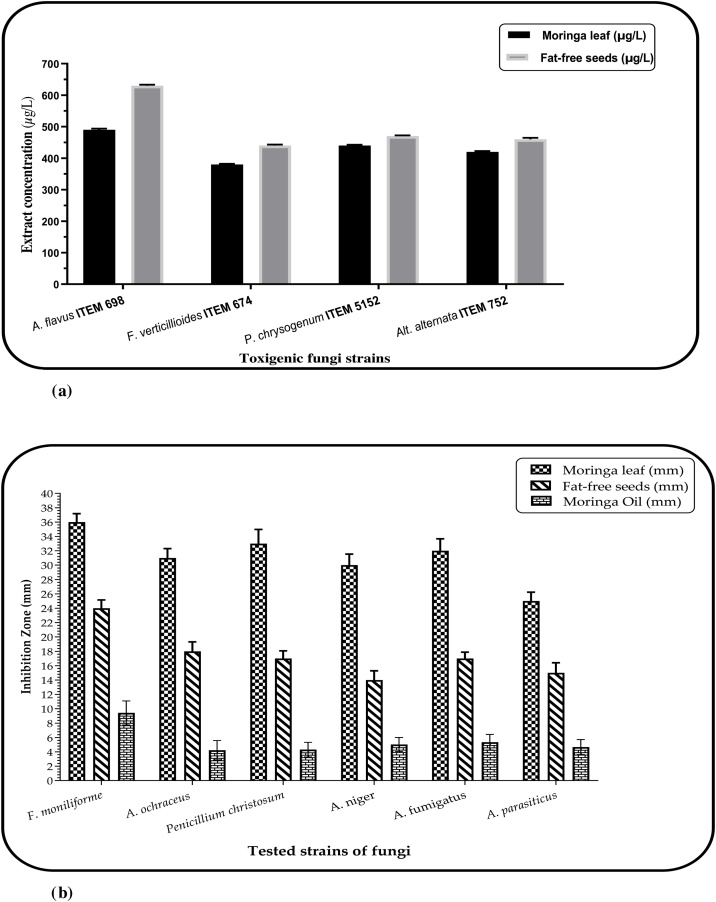
(a): The minimal fungicidal concentration of moringa leaves and fat-free seeds extracts against mycotoxigenic strains. (b): Inhibition zone diameters (mm) of moringa leaves, oil, and fat-free seeds extracts measured on agar media against food-fungal strains.

Nevertheless, the effect of Moringa extracts against several toxigenic strains of fungi manifested a high effective impact, particularly for the MLE. The estimation of the minimal fungicidal concentration of Moringa extracts in liquid media appeared close in some fungi strains for leaves and seed extracts (Fig. 1a). However, the determination of inhibition zone impact using agar media showed a tangible difference between the leaf and seed extract (Fig. 1b). Again, *Fusarium* fungi manifested more sensitivity for the treatment using the MLE extract compared to other strains of applied fungi. Moreover, the MLE was still able to inhibit the growth of four *Aspergillus* fungi (*A. ochraceus; A. niger; A. fumigatus; A. parasiticus*), which deems more dangerous fungi. As the strain of *A. parasiticus* has the ability for aflatoxins production, the inhibition of its growth using the MLE may lead to less concentration of aflatoxins secretion in their media of growth.

### 3.6 Application of Moringa extracts for peanuts protection

**Fig. 2:**
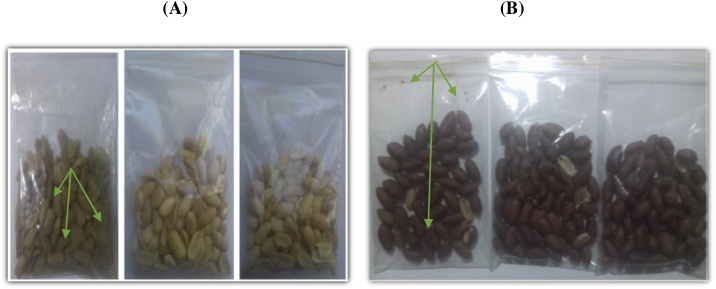
effect of moringa film composite as a protective agent against toxigenic *Aspergillus* infection. ● The 1st bag of each treatment positive control (with fungi), 2nd bag was the negative control (without fungi), and 3rd bag of peanut was the coated seeds for a group (A) and (B), respectively. ● Group (A) was peanut seeds with peels; Group (B) was peanut seeds without peels. ● The Arrows point out the growth and hypha of fungi in the control treatment.

Using the MOO as a carrier for the MLE to form micro-emulsion (W/O) utilized for peanut coating manifested a protective function against fungal infection by an applied strain of toxigenic *Aspergilli*. The particle size of the emulsion was ranged from 282 to 371 nanometers. It was noticed that the coated inoculated samples of peanuts were more resistant than the control ones. However, the protective effect was more clearance for the shield-peanut that had the red-peel coat (Fig. 2). The Data represent a high capacity for the effect of Moringa emulsion, which is composited from Moringa extracts (MLE and MOO). The red-shield peel of peanut seeds may possess antioxidant activity, as a seed defense, that increases their protection against inoculated fungi.

The results expressed the high efficacy of Moringa emulsion in protecting both peanut shielded and unshielded seeds. The CFU counts of inoculated fungi for infected control and infected coated shield-seeds were 6.8*10^4^ and 9 CFU/Kg, respectively. While, The CFU counts of inoculated fungi for infected control and infected coated unshielded seeds were 9.4*10^5^ and 14 CFU/Kg, respectively. These results have reflected the efficiency of applied emulsion for limiting the contamination by *Aspergillus* toxigenic strain during the storage and handling of ready-to-eat seeds. For aflatoxin B_1_ (AFB_1_) contamination, control seeds were recorded with 480 ng and 945 ng/kg AFB_1_/kg of peanut seeds, for the shield and unshielded seeds, respectively. The film-coated seeds of shielded and unshielded peanuts were recorded with a non-detectable aflatoxins amount.

## 4 Discussion

Because of the bioactive contents in various parts, Moringa trees have considerable pharmacological, nutraceutical, and antioxidant properties [44,45]. Where these phytoconstituents were reported to be dense in plant leaves [46]. The proximate analysis of leaves showed high carbohydrates, fiber, and protein contents (Table 1). The bioactive molecules of the Moringa plant could belong to phenolic, flavonoids, minor constituents, or kinds of carbohydrates. These bioactive possess varied characteristics to act as nutritional, antimicrobial, and anti-mycotoxigenic functions [5,7]. The fiber fraction manifested significant quantities of cellulose, hemicellulose, and lignin in leaves. Regarding their content of total phenolic, flavonoids, and antioxidant potency, the MLE possesses bioactive molecules that support its antimicrobial potency that help in defending against pathogens [15,47].

The presence of vitamin E fractions (tocopherol and tocotrienol) and sterols awarded Moringa oil its stability due to their bioactivities, particularly against microbial spoilage [19]. It also gives a safety and shelf-life stability as well as a long storage period [6,30,47]. Plant extracts with a distinct content of bioactive ingredients showed antimicrobial potency for both spoilage-causing bacteria and fungi [5,7,28]. This potency was reported for both *in vivo* [23,38,39] and *in vitro* [26,48,49] implementations. Also, it considers pretty when plant extracts possess activities to inhibit toxin-producing fungi or the ability to reduce their mycotoxins [24,29,38]. The impact of these extracts is mainly joined to their antioxidant potency [47]. This property affects the oxidative stress that forces the fungal cells to produce their toxins on products and change the fungal metabolism to be safer [7,8]. Phenolic compounds, including the flavonoids, sourced from plant extract exceedingly acting this function [34,50].

The ML and the seeds are considered rich in antioxidants and phenolic compounds [16,26,51]. Also, the seeds were distinguished by their content of enzymes and bioactive proteins [12,18,52], which were subsequently transferred to their extract. In this regard, the authors planned to apply the MOO supplemented by MLE as a biofilm to increase the safety and shelf life extension of peanuts as a type of oilseeds. the MLE was previously used as a marinade solution to protect fish-fillet against aflatoxigenic fungi and reduce aflatoxins content during storage [53]. At that experiment, saline may support or reduce the efficiency of the MLE to reduce mycotoxin occurrence. Contrarily, the MLE encapsulated by the MOO was applied in this investigation. Current oil is differentiated as it was obtained by cold-press techniques, which give a more chance for bioactive presence.

Regarding the application of oil-based film, the results reflect enhancement for the reduction of aflatoxins-contents. Again, the inoculated strain of *A. flavus* ITEM 698, which is a high producer of aflatoxins, was recorded by CFU-count inhibition in coated peanuts compared to the control. Moringa oil is characterized by its high Oleic and Palmitic acid content, which are known functionalized by antimicrobial activity [54,55]. Also, the calculated oxidizing-ability (COX-value) of oil reflects high oil stability against auto-oxidation. This could save its application from being refused in food applications due to chemical changes during storage time. The tocols content of Moringa oil was recognized by a valuable amount of tocopherols and tocotrienols (Table 4). Moreover, the sterols content shows stigmasterols as the main fraction followed by the B-sitosterols, where these components were reported as high antimicrobial activities [56]. Furthermore, the MLE was distinct by their phenolic and flavonoid content, besides their antioxidant activity (Table 5). The MIC and MFC values of the extract were higher compared to the seed extract, the same efficiency was recorded for the diffusion assay against toxigenic fungal strains. These results give evidence for the antimicrobial potency of the MLE, which bolstered their implementation in food applications.

The presence of such components in the film composition that consists of oil and leaves-extract assisted it to be an effective shield for seeds against fungal contamination. The film-coated seeds of peanuts were recorded more resistance for the contamination by the mycotoxigenic Aspergillus strain during the storage. After three months of storage for inoculated seeds, the film-coated seeds were highly resistant with a very low CFU fungal count. Moreover, it was recorded with an absence of aflatoxins contamination. In comparison to a previous study by Shehata et al. [30] some oils possessed special characteristics that make them suitable to apply as a film-coating and carry a source of bioactive components. This type of coating was effective against mycotoxigenic fungi by application to protect oil-seeds. Consequently, Moringa oil will make a difference in food applications and processing, particularly in the safety and hygiene handling of the final food products.

## 5 Conclusions

Moringa has nutraceuticals and antioxidants due to the bioactivity of oil and leaves extract. These constituents have a significant antimicrobial potency that increases shelf-life stability by their applications. The MOO was distinct with tocopherols and sterols content, which was previously known by antimicrobial activity. As well, the leaves extract was recorded contains considerable phenolic compounds. The MLE efficacy for diffusion assay and MFC values showed significantly. The distinguished characterization of Moringa extracts nominated them to apply in seed coating against toxigenic fungi. A compositing film of Moringa oil and its leaves extract was implemented in peanuts seed protection against the aflatoxigenic strain of *Aspergillus* fungi. The results manifested the film-coating capacity to stop aflatoxin secretion and the strong limitation of fungal growth of inoculated seeds. Moreover, AFB_1_ was non-detectable in coated seeds; comparing to 480 ng/Kg and 945 ng/Kg in inoculated control shield and unshielded seed.

## Author contributions

ANB and YHA, Conceptualization; SMA and AGA, Data curation; ANB and AGA, Formal analysis; SMA and ANB, Investigation; ANB and YHA, Methodology; AGA and ANB, Project administration; YHA, Resources; ANB, Supervision; YHA and ANB, Validation & Visualization; ANB, Writing - original draft; AGA and SMA, Writing - review & editing.

## Conflict of interest

The authors declare no conflict of interest.

## Declaration of Competing Interest

The authors report no declarations of interest.
